# Flavonoid Extracts from Lemon By-Products as a Functional Ingredient for New Foods: A Systematic Review

**DOI:** 10.3390/foods12193687

**Published:** 2023-10-08

**Authors:** Lorena Martínez-Zamora, Marina Cano-Lamadrid, Francisco Artés-Hernández, Noelia Castillejo

**Affiliations:** 1 Department of Food Technology, Nutrition and Food Science, Faculty of Veterinary Sciences, University of Murcia, 30071 Espinardo, Murcia, Spain; 2 Postharvest and Refrigeration Group, Department of Agronomical Engineering and Institute of Plant Biotechnology, Universidad Politécnica de Cartagena, 30203 Cartagena, Murcia, Spain; marina.cano@upct.es (M.C.-L.); fr.artes-hdez@upct.es (F.A.-H.); 3 Department of Agricultural Sciences, Food, Natural Resources and Engineering, University of Foggia, Via Napoli 25, I-71122 Foggia, Italy

**Keywords:** hesperidin, naringenin, food loss, lemon skin, co-products

## Abstract

This systematic review seeks to highlight, from the published literature about the extraction and application of lemon by-products rich in flavonoids, which works use environmentally friendly technologies and solvents and which ones propose a potentially functional food application, according to the Sustainable Development Goals (SDGs). WoS and SCOPUS were used as scientific databases for searching the documents, which were evaluated through 10 quality questions according to their adherence to our purpose (5 questions evaluating papers devoted to lemon flavonoid extraction and 5 concerning the application of such by-products in new foods). Each question was evaluated as “Yes”, “No”, or “does Not refer”, according to its adherence to our aim. The analysis reported 39 manuscripts related to lemon flavonoid extraction; 89% of them used green technologies and solvents. On the other hand, 18 manuscripts were related to the incorporation of lemon by-products into new foods, of which 41% adhered to our purpose and only 35% evaluated the functionality of such incorporation. Conclusively, although the bibliography is extensive, there are still some gaps for further investigation concerning the extraction and application of lemon by-products to reduce food losses in an environmentally friendly way and the possible development of new functional foods, which must be performed following the SDGs.

## 1. Background

The Food and Agriculture Organization (FAO) reports that approximately one-third of the global production is lost or wasted at some stage of the food chain [1]. FAO’s future challenges for 2050 are to reduce food waste by 50%, one of the SDGs. Circular economy has been seen as the principle of a society-driven and ‘zero waste’ economy, with waste as raw materials.

According to FAOSTAT, the global production of lemon was around 21.4 million tons in 2020 [2]. Although it depends on the variety, the juice yield of these citrus reaches values of 38–41% [3]. Peels, pulp, seeds, and pomace, which constitute approximately 50% of the fresh fruit, are some of the wastes generated by citrus processing and consumption [3]. Similar values are being obtained in our own laboratories (45%) in the Primofiori variety, while they decreased to 28% in varieties with a thicker albedo, such as the Verna variety (unpublished data). This means that, according to the variety, between 55 and 72% of a lemon is directly wasted after squeezing. In global figures, this leaves between 11.8 and 15.4 million tons of food losses per year with high nutritional value that can be recovered as sources of bioactive compounds, essential oils, and fiber.

In this sense, these food losses are currently used by flavor and extraction companies to obtain essential oils and fiber for their application as flavorings and odorizing or emulsifying agents. Linked to the rise in demand for a healthy diet and the pursuit of the SDGs, the extraction and purification of bioactive compounds from food by-products have exponentially increased in the last two decades.

In this perspective, sustainability, well-being, and health are currently popular topics in the food industry. ‘Clean label’ goods or components appeal to both consumers and food manufacturers [4,5,6]. This indicates that people are interested in a variety of green-processed foods and ingredients, including nutraceuticals, bioactive chemicals with health-promoting qualities, and non-thermal green solvents. The technical and functional properties of the bioactive compounds derived from fruit and vegetable by-products allow them to be incorporated into other food matrixes to improve their nutritional, functional, and sensory qualities [5,7]. Additionally, the use of bioactive chemicals from fruit and vegetable by-products has been previously categorized as a potential green element for the cosmetic and pharmaceutical sectors, generating several products aimed at niche markets such as athletes [8].

In fact, regarding European regulations, no health claim is yet authorized for ‘antioxidants’ and ‘flavonoids’ from lemon. The reason for the negative opinion from the EFSA is the non-compliance with the regulation based on the scientific evidence assessed. The claimed effect of this food has not been substantiated. There are currently no authorized health claims for lemon and its constituents in the European Union.

Consequently, several reviews have been published so far related to this topic, like the one recently published by Magalhães et al. [9], who widely exposed the major compounds found in lemons, their main extraction technologies, and their applications in food preservation. In this sense, as a review of the extensive bibliography on the topic has already been performed, the aim and novelty of the present systematic review is to account for which of the literature published on the topic is truly adapted to these SDGs, uses environmentally friendly technologies and solvents, and develops a potentially functional food application of flavonoids, the main bioactive compounds extracted from lemon by-products.

## 2. Lemon By-Products and Their Functional Quality

According to the structure of the lemon fruit, it is divided into the albedo, which is the main source of fiber (pectin and cellulose), the flavedo, which is rich in essential oils and pigments, and the pulp, where the juice, rich in water and nutritional and functional compounds (citric acid, ascorbic acid, minerals, and flavonoids), is obtained.

The albedo is the bitter white layer that surrounds the juicy pulp of the fruit. It contains pectin, fiber, and other nutrients [10]. Because of its bitterness, in the lemon processing industry, the albedo is usually removed from the fruit as a non-edible part. For lemon essential oil production, the processors use a method called cold-pressing, in which the lemon peels are soaked in water and then pressed to extract the oil. This method produces a high-quality oil with a fresh, citrusy fragrance, which is used in perfumes, cosmetics, and food flavorings.

In fact, this bitterness is caused mainly by the presence of phenolic compounds, which include phenolic acids and flavonoids. Hydroxycinnamic (chlorogenic, caffeic, ferulic, sinapic, and p-coumaric acids) and hydroxybenzoic acids (protocatechuic, p-hydroxybenzoic, vanillic, and gallic acids) have been identified in lemon peels [11]. Nevertheless, flavonoids such as hesperidin (59% of the flavanone content) and eriocitrin (35.6% of the flavanone content) are the most concentrated in the albedo. Furthermore, other minor flavanones such as didymin, naringin, neoeriocitrin, neohesperidin, narirutin, eriodictyol, and naringenin have been identified. Also, the favones diosmetin, diosmin, luteolin, vicenin, chrysoeriol, apigenin, and sinensetin and the flavonols quercetin, limocitrin, limocitrol, rutin, and kaempferol are also present in lemon by-products [9,10].

For this reason, and due to their bioactivity, lemon flavonoids have a good potential to be extracted and applied in new functional foods. Flavonoids are polyphenolic compounds with a broad range of biological activities, including antioxidant, anti-inflammatory, and anticancer effects. Hence, its consumption has been associated with the preventive effects of chronic diseases by avoiding inflammation and oxidative stress. The combination of these bioactive compounds and dietary fibers in lemon fruits makes them a valuable addition to a healthy diet and lifestyle.

In this respect, the dietary fiber contained in the lemon albedo includes gums, pectins, glucans, and some polysaccharides as insoluble fibers, while cellulose, hemicellulose, and lignin are soluble fibers. Particularly, pectin is the major component of such fiber, and although it cannot be digested by the human intestine, our microbiota is able to assimilate and convert it into beneficial metabolites [12].

In addition, the flavedo is rich in volatile compounds and essential oils. For instance, d-limonene is the main essential oil of the lemon flavedo, followed by β-pinene, γ-terpinene, α-pinene, sabinene, myrcene, and α-thujene, among others [10]. However, in the present review, we will focus on the bioactivity of the main compounds cited above.

## 3. Methods

**Figure 1:**
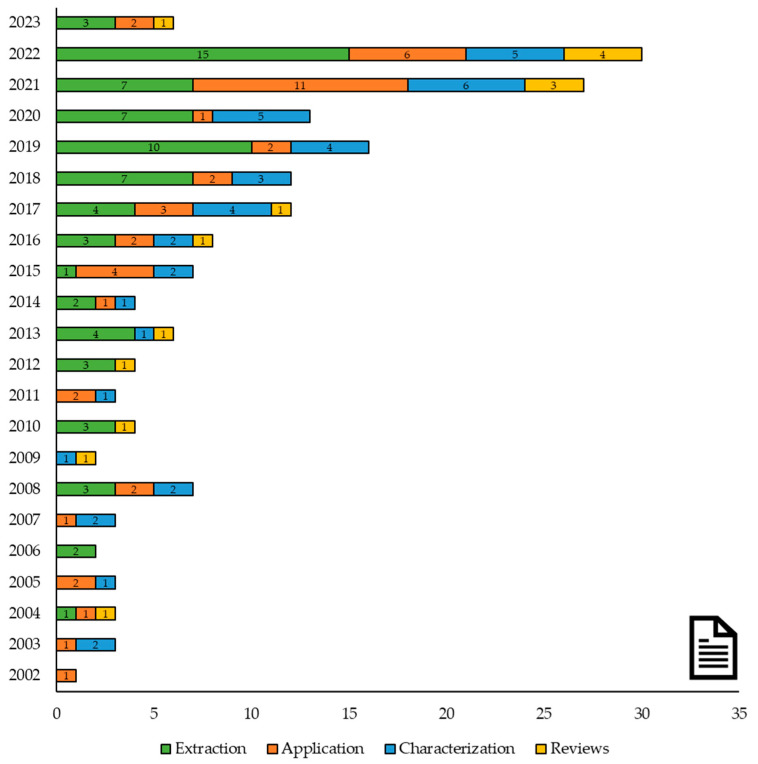
Number of research papers and reviews related to lemon by-products published in the last twenty years, according to WoS and SCOPUS (*n* = 176).

WoS and SCOPUS were used as scientific databases for searching documents. The terms “lemon”, “*Citrus limon*”, “co-products”, and “by-products” were used as keywords. Other search words used were “extraction”, “flavonoids”, “ultrasound-assisted extraction”, “microwave-assisted extraction”, and “enzymatic-assisted extraction” for manuscripts related to the extraction of lemon by-products. The terms “application”, “food”, “juice”, and “beverage” were used for manuscripts related to the application of lemon by-products in new food matrixes. First, a description of the total literature found in reference to “lemon”, “*Citrus limon*”, “co-products”, and “by-products” published in the last twenty years was carried out, including the number of reviews in reference to this topic (Figure 1).

From the bibliography found and described in the raw analysis, the inclusion criterion for our systematic review was “original studies included in JCR-SCI journals’”. The exclusion criterion was “studies non-included in JCR-SCI journals, books, and reviews”. The title and abstracts of the documents found were analyzed and classified depending on their significant interest using Microsoft Excel for the data curation. The potential scientific papers were subjected to a comprehensive analysis, in which all the papers were checked for the inclusion quality criteria. The 10 following questions were used as quality criteria (5 of the questions (from 1 to 5) were related to the evaluation of the quality of the manuscript related to the extraction of lemon by-products, and the other 5 (from 6 to 10) were related to the evaluation of the quality of the manuscript related to the application of such lemon by-product extracts): (Q1) Does the article include an experimental design (response surface methodology)?; (Q2) Does it use green solvents for the extraction?; (Q3) Does it use green technologies for the extraction?; (Q4) Does it extract flavonoids?; (Q5) Do the authors validate/characterize their extract?; (Q6) Do the authors incorporate these flavonoids into food?; (Q7) Do the authors encapsulate the extract?; (Q8) Do the authors conduct a shelf-life study with the new food?; (Q9) Do the authors compare it to a control sample?; (Q10) Do the authors evaluate the functionality of the enriched food?

**Figure 2:**
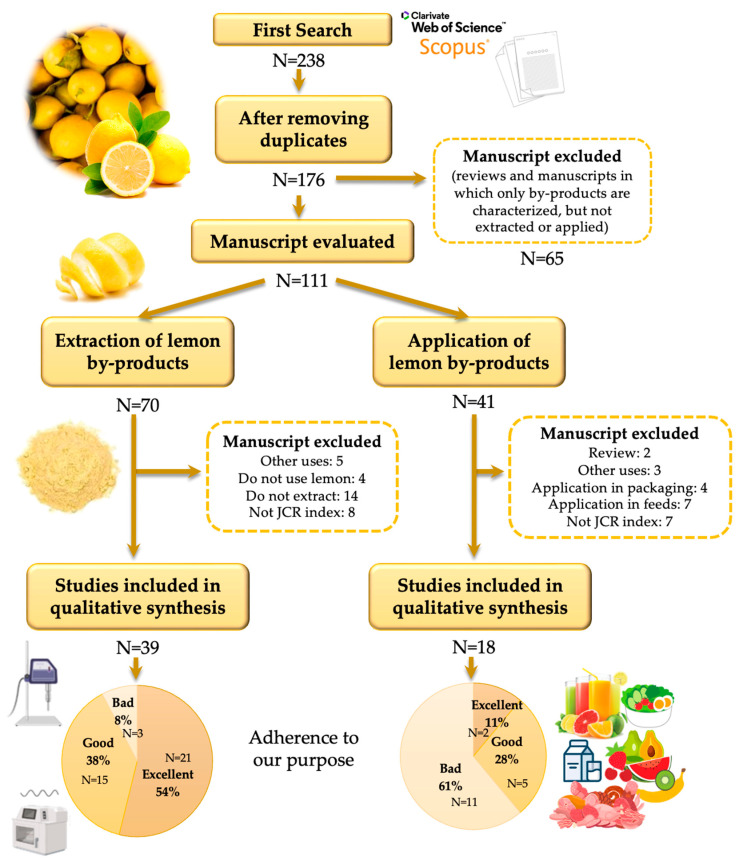
PRISMA flow diagram followed, studies selected, and classification in reference to the proposed quality criteria.

Each query was evaluated as “Yes”, “No”, or “does Not refer”. The frequency of “Yes” responses for each one was used to determine the quality and reproducibility of this study. The works were arranged into three categories: excellent (>70% “Yes” responses), good (50–69% “Yes” responses), and bad (<50% “Yes” responses), according to their adherence to our purpose. The PRISMA flow diagram followed, and the results obtained in this systematic review are shown in Figure 2.

## 4. Results and Discussion

As shown in Figure 2, of all the studies found in the literature, 39 were included in the qualitative analysis for extraction, of which 92% suited well to our main goal, while the remaining 8% did not. Moreover, 18 scientific studies were included in the analysis related to the application of lemon by-products, of which 39% were in good compliance with our purpose.

### 4.1. Flavonoid Extraction

Table 1 shows the results obtained from the qualitative analysis carried out in the 39 scientific studies found.

The studies reviewed in this list explore various methods for the extraction and utilization of valuable components from citrus peels and by-products.

Due to the SDGs, petrochemical solvents have been so far replaced by green solvents in many recent studies. Green solvents must be environmentally sustainable and are characterized by high-quality products with fewer by-products produced during processing and low toxicity. The main green solvents are ionic liquids, deep eutectic solvents (DESs), polyethylene glycol (PEGs), ethyl lactate, water, supercritical fluids, alcohols (ethanol), esters (ethyl lactate and ethyl acetate), and terpenes [52,53,54]. Other solvents, such as xylenes, methanol, tetrahydrofuran, DMSO, chlorobenzene, thiophene, and diphenyl ether, are still widely used and sometimes considered to be green solvents, although little evidence of this has been found [55].

As previously described by Artés-Hernández et al. [4,5,6], some examples of green technologies are ultrasound-, microwave-, and enzymatic-assisted extraction, supercritical or subcritical fluids, and pressurized liquids, which are the most widely used in the studies reviewed. Another green technology is cold pressing, a fast, inexpensive, solvent-free, and environmentally friendly process, but its yield is often lower than that of solvent extraction [22].

Phenolics and pectins are commonly targeted for extraction, with enzyme-assisted extraction showing promise as an effective method. Lemon peels have been shown to be useful to produce pectin-derived oligosaccharides and polyphenol extracts, as well as bioethanol [19]. Other studies have explored the use of lemon peel waste for the removal of heavy metals from wastewater and the production of humic acid [29]. Novel approaches include the use of ultrasound- and microwave-assisted extractions, as well as the integration of pressurized liquid and in-line solid-phase extractions for the simultaneous extraction and concentration of phenolic compounds [42]. Overall, the studies suggest that utilizing citrus peels and by-products can be an effective way to reduce waste and extract valuable components for various applications that are going to be summarized below.

Li et al. [13,14] investigated the extraction of phenolics from citrus peels through two different methods: solvent extraction [14] and enzyme-assisted extraction [13]. Both methods resulted in high yields of phenolic compounds. However, the enzyme-assisted method was found to be more efficient and faster compared to the solvent extraction method [13]. Masmoudi et al. [15] studied the effect of different extraction methods on the antioxidant properties of citrus peels. They found that ethanol and water were the best solvents for extracting phenolics from citrus peels. The study also showed that microwave-assisted extraction had a higher extraction efficiency compared to traditional methods.

These studies highlight the potential value that can be generated from citrus peels and by-products. Efficient extraction methods can be used to obtain valuable bioactive compounds with potential health benefits and industrial applications using green solvents, mainly ethanol and water, and novel green technologies such as enzymatic-, ultrasound-, pressurized-, pulsed electric field-, or microwave-assisted extractions. Moreover, utilizing citrus peels can significantly decrease environmental pollution caused by the disposal of waste citrus materials and is an important source of flavonoids to be applied to new foods, as shown below.

### 4.2. Application

The results obtained from the qualitative analysis carried out for the 18 scientific studies found are shown in Table 2. The works listed in this collection focus on the use of citrus by-products, particularly lemon, in various food applications. These applications include the use of citrus fibers and albedo in meat products [56], the preparation and characterization of osmodehydrated fruits [57], the incorporation of citrus fibers in fermented milk containing probiotic bacteria [58], as well as cake or bakery products [51,59,60]. Other studies examine the potential of citrus by-products as fat replacers in chicken patties [61], as antioxidants in food flavorings, and as a means of improving the bio-accessibility of polyphenols in salad dressings [62]. Additionally, several works evaluate the efficacy of antioxidant extracts from lemon by-products in preserving the quality attributes of minimally processed radish [63].

In addition, lemon juice has been used as a natural acidifying agent, e.g., Banerjee et al. [64] used lemon juice instead of HCl in the valorization of mango peels to lower the pH to 2.5 and recover pectin. Furthermore, lemon peels have been studied as removers of heavy metal ions (Fe^2+^, Zn^2+^, and Mn^2+^) in wastewater [65]. Lemon peels in a 0.1 M HCl solution were able to reach a value of 55.19% for Mn^2+^ desorption and 37.24% for Zn^2+^, while for Fe^2+^, the highest value of 25.82% was achieved in a 0.1 M HNO_3_ solution.

Overall, these studies demonstrate the potential of citrus by-products as functional ingredients in various processed food products, such as fruits, vegetables, dairy, bakery, and meat products. For that reason, this topic must be further investigated with the goal of incorporating these new ingredients as food preservatives to recover part of the produced food discards with potential health benefits, which must be validated by international agencies (such as the EFSA in Europe or the FDA in the USA).

**Table 2:** Application of citrus by-products and their potential benefits.

Enriched Product	Q6	Q7	Q8	Q9	Q10	Main Findings	Adherence to Our Purpose	Ref.
Fresh British-style pork sausages	No	No	No	Yes	No	7% of citrus fiber extract reduced the shrinkage and the cooking loss, increased lightness (L*), and maintained their antioxidant effect as well as the overall acceptance of cooked sausages	Bad	[56]
Swedish-style beef meatballs	Yes	No	Yes	Yes	No	Citrus extracts reduced the rancidity of meat products by 50%	Good	[66]
Osmodehydrated fruits	No	No	Yes	Yes	No	Microbially stable for 3 months at 4 °C	Bad	[57]
Meat emulsion systems	No	No	No	Yes	No	Lemon albedo addition did not change the flow properties and improved the texture, acting as a source of fiber	Bad	[67]
Fermented milk	No	No	Yes	Yes	No	Enhanced survival of the tested probiotic bacteria and bacterial growth, maintaining the acceptability	Bad	[58]
Dough and Mantou (steamed bread)	Yes	No	No	Yes	No	3 or 6 g per 100 g of flour produced acceptable Mantou with higher antioxidant capacity and total phenolic content	Bad	[68]
Frankfurters	Yes	No	No	Yes	No	Incorporation of shaddock albedo increased hardness and decreased chewiness; hence, it can be a potential emulsifier	Bad	[69]
Oat–fruit juice mixed beverage	Yes	No	Yes	Yes	No	Antimicrobial effect against *Salmonella typhimurium* and *E. coli* of all the extracts at 5 °C	Good	[70]
Food flavoring	No	No	Yes	No	No	Extracts thermally stable and safe	Bad	[71]
Lemon oil	No	No	Yes	Yes	No	Nanoemulsions of lemon and fish oil by-products (8% lemon oil, 2% fish by-product oil, 10% surfactant, and 27.7% cosurfactants) inhibited 7 Gram-positive and 7 Gram-negative bacterial strains	Bad	[72]
Biscuits	Yes	No	No	Yes	No	Higher phenolic content and antioxidant activity with suitable acceptability	Bad	[59]
Cake	No	Yes	No	Yes	No	Greater acceptability by a 10% fat replacement, which presented an increase in dietary fiber	Bad	[60]
Sunflower oil	Yes	No	No	Yes	Yes	The antioxidant effects of citrus extracts (mandarin, orange, and lemon) were comparable to BHT, with lemon being the most antioxidant against lipid oxidation	Good	[73]
Ultra-low-fat chicken patties	Yes	No	No	Yes	Yes	Lemon albedo decreased fat and cholesterol content, increased cooking yield, and showed good acceptability	Good	[61]
Salad dressing	Yes	No	Yes	Yes	Yes	Increased the bioaccessibility of hydroxycinnamic acids and flavonols by 0.3- to 5.8-fold	Excellent	[62]
Fresh-cut radish	Yes	No	Yes	Yes	No	Lower color variation and mesophilic aerobic count, proving a shelf-life of 7 days at 3 °C	Good	[63]
Cake	Yes	Yes	Yes	Yes	No	Nanoencapsulated lemon reports lower antioxidant activity and yield compared to orange; no significant difference in sensory quality or acceptability	Excellent	[51]
Chicken emulsion	No	No	No	Yes	No	2% added citrus peel fiber reported the best quality (viscosity, cooking loss, and emulsion stability)	Bad	[74]

Q6: Do the authors incorporate these flavonoids into a food?; Q7: Do the authors encapsulate the extract?; Q8: Do the authors conduct a shelf-life study with the new food?; Q9: Do the authors compare to a control sample?; Q10: Do the authors evaluate the functionality of the enriched food?

## 5. Future Perspective and Main Conclusions

The main conclusion of the present systematic review is that almost 90% of the selected publications related to the extraction of bioactive compounds from lemon by-products used environmentally friendly technologies and solvents. They greatly contributed to the optimization of the extraction of the bio-compounds, which are mainly present in the flavedo and albedo of lemon peels, the main food discards of these citrus. Nevertheless, further research is still necessary relating to the incorporation of these lemon extracts into potential new functional foods, especially concerning the assessment of the functionality and direct benefits produced by the consumption of such new foods enriched in flavonoids from lemon by-products, which have been shown to be an important source of health-promoting compounds. In addition, further research is also needed regarding green technologies to reduce energy in the by-product’s revalorization process by applying an efficient and environmentally friendly solvent extraction method.

## Figures and Tables

**Table 1:** Non-thermal extractions of citrus by-products.

By-Product Characteristics	Q1	Q2	Q3	Q4	Q5	Optimum Conditions of Extraction	Adherence to Our Purpose	Ref.
Lemons (*Citrus limon* cv. *Meyer*), lemon (*C. limon* cv. *Yenben*) peels (epicarp and mesocarp), frozen and milled (Ø: <1 mm)	No	Yes (Water)	Yes (Enzymatic)	Yes	Yes	Celluzyme MX 1.5% at 50 °C	Excellent	[13]
Lemons (*Citrus limon* cv. *Meyer*), lemon (*C. limon* cv. *Yenben*) peels (epicarp and mesocarp), frozen and milled (Ø: <1 mm)	No	Yes (Water and ethanol)	Yes (Temp.)	Yes	Yes	85% ethanol and 80 °C	Excellent	[14]
Lemon (*Citrus limon* L.) peels and pulps, freeze-dried and milled (Ø: 0.25 mm)	Yes (Central composite design)	Yes (Ethanol and acidified date juice)	Yes (Stirring)	No	Yes	84.34 °C for 3 h 34 min, and pH 2.8	Excellent	[15]
Lemon (*Citrus limon* L.) peels and pulps, freeze-dried and milled (Ø: 0.25 mm)	No	Yes (Water with acidified date juice)	Yes (Stirring)	No	Yes	pH 3.5, 45% sucrose, and 0.1% calcium to improve the gelling properties of the extracted pectin	Good	[16]
Lemon (*Citrus limon* L.) peels and pulps, freeze-dried and milled (Ø: 0.25 mm)	No	Yes (Water with acidified date juice)	Yes (Stirring)	No	Yes	84.34 °C for 3 h 34 min, and pH 2.8	Good	[17]
Lemon peels (Ø: <3 mm)	No	Yes (Water)	Yes (Enzymatic)	No	Yes	Water at 160 °C in autohydrolysis with two membrane filtration stages (diafiltration and concentration)	Good	[18]
Lemon peels (Ø: <7 mm)	No	Yes (Water)	Yes (Enzymatic)	No	Yes	2:1 ratio (lemon peels:water) at 37 °C with 1.95 mg β-glucosidase, 2.21 mg pectinase, and 1.82 mg celullase using steam explosion	Good	[19]
Lemon peels	No	Yes (Water)	Yes (Enzymatic)	No	Yes	From 7.5 to 24 h enzymatic hydrolysis and membrane processing (filtration and concentration)	Good	[20]
Lime peel (*Citrus limonium* cv. *Colima*) squares (5 mm × 5 mm)	No	Yes (Citric acid–sodium citrate)	Yes (Enzymatic)	No	No	1:2.5 ratio (peels:solvent) + 0.1% cellulase enzyme at 50 °C for 3 h	Bad	[21]
Lemon (*Citrus limon* L. var. Kütdiken) seeds	No	Yes –	Yes (Cold-pressed)	No	Yes	High-quality oils from seeds with a moisture content of 12% and cold-pressed (screw rotation speed of 30 rpm, outlet matrix of 10 mm, and oil outlet temp. of 40 °C)	Good	[22]
Yuzu (*Citrus junos*) peels and seeds, dried and milled (Ø: <0.71 mm)	Yes (Box–Behnken)	Yes (CO_2_)	Yes (Supercritical fluids)	No	Yes	200.54 bar, 46.28 °C, and 34.98 g/min flow rate	Excellent	[23]
Cold-pressed EOs derived from lemon industrial processing	No	Yes (Water)	Yes (Cold-pressed)	No	Yes	Hydrodistillation for 3 h	Good	[24]
Lemon peels, membranes, and seeds, freeze-dried and milled (Ø: <1.4 mm)	No	Yes (Water)	Yes (MW)	Yes	Yes	360 W for 5 min (72 kJ/g)	Excellent	[25]
Lemon (*Citrus limon* L.) peels, membranes, and seeds, freeze-dried and milled (Ø: 1.4, 2, 2.8 mm)	Yes (Box–Behnken)	Yes (Water, hot water, and ethanol)	Yes (US, temp., and stirring)	Yes	Yes	US: 35–45 min, 48–50 °C, 150–250 W Temp.: 95 °C for 15 min	Excellent	[26]
Cold-pressed meals of lemon (*Citrus limon* L.) seeds, dried	No	Yes –	Yes (Cold-pressed)	Yes	Yes	150 °C for 30 min and cold-pressed (30 rpm, 10 mm die, and 40 °C)	Excellent	[27]
Domestic house solid waste lemon peel, milled and dried	No	No	Yes (HMIM)	Yes	Yes	80 °C for 3 h in a water bath, cooled, and filtered	Good	[28]
Lemon (*Citrus limon* L.) peels, dried and milled (Ø: 0.6–1.5 mm)	No	Yes (Water)	Yes (Subcritical fluids)	No	Yes	10 g at 1500 psi, 200 °C, 15 min	Good	[29]
Lemon (*Citrus limon* L.) peels, chopped (Ø: 10–30 mm)	No	Yes –	Yes (Pulsed electric fields)	Yes	Yes	5 bars, 3.5 kV/cm, 30 pulses of 30 µs	Excellent	[30]
Lemon seeds, dried and milled (Ø: 0.25–0.425 mm)	No	Yes (CO_2_)	Yes (Supercritical fluids)	No	Yes	First separator: 300 bar and 40 °C Second separator: 20 bar and 15 °C	Good	[31]
Lemon peels, dried and milled (Ø: 0.787 mm)	Yes (Box–Behnken)	Yes (Ethanol–water)	Yes (Homogenizer)	Yes	Yes	0.1 g mixed in 33.62% ethanol for 1.282 min at 5007 rpm	Excellent	[32]
Sweet lemon peels, dried and milled (Ø: 0.4 mm)	Yes (Box–Behnken)	Yes (Ethanol–water)	Yes (MW)	No	Yes	700 W, 3 min, and pH 1.5	Excellent	[33]
Lemon (*Citrus limon* L.) peels, dried and milled (Ø: <0.15 mm)	No	Yes (Water or ethanol)	Yes (Temp.)	Yes	Yes	10 g in 200 mL ethanol at 60 °C for 2 h	Excellent	[34]
Lemon (*Citrus limon* L.) peels, blanched and frozen	Yes (Box– Behnken)	Yes (Citric acid)	Yes (Ohmic heating)	No	Yes	8.7:1 (solvent:sample) ratio for 58.4 min and voltage gradient of 14.2 V/cm	Excellent	[35]
Persian lime (*Citrus latifolia*) seeds, dried	No	Yes (Phosphate buffer)	Yes (Enzymatic)	No	Yes	Alcalase, Protamex, and Neutrase mixed enzymes (1:1:1), pH 8.0, 50 °C	Good	[36]
Lemon peel pomace (Ø: 0.177 mm)	No	Yes (Citric acid and phosphate buffer)	Yes (Enzymatic)	No	Yes	0.45% xylanase, pH 5.0, rate (solid:liquid) 1:20 at 60 °C for 1.5 h at 30 rpm	Good	[37]
Frozen lemon peels and pulps	No	Yes (Ethanol)	Yes (HPH)	No	Yes	20 MPa to alcohol-insoluble residue	Good	[38]
Lemon (*Citrus limon* L. *Osbeck*) peels	No	Yes (Water and ethanol–water)	Yes (US and MW)	Yes	Yes	Ethanol:water (50:50), US at 70 °C for 30 min	Excellent	[39]
Lemon peels, dried and milled (Ø: ~0.1 mm)	Yes (Central composite design)	Yes (Deep eutectic solvents)	Yes (Stirring)	Yes	Yes	55% (sample/solvent), 13 mL/g and 36 min in deep eutectic solvents	Excellent	[40]
Lemon peels	Yes (Box–Behnken)	Yes (Lactic acid-based automatic solvent)	Yes	Yes	Yes	1.5 h, 46% water, and 5 g of peel	Excellent	[41]
Lemon peels, dried and crushed (Ø: >1 mm)	No	Yes (Ethanol and water)	Yes (PLE-SPE)	Yes	Yes	Sepra™ C18-E columns, in water–ethanol, pH 6–7, temp. 50–70 °C	Excellent	[42]
Lemon peels, dried	Yes (Uncertainty analysis)	Yes (Water)	Yes (Steam distillation)	No	Yes	Lemon peel oils report better results compared to normal diesel in all aspects, except for NOx emissions. A content of 10% water in lemon peel oils improves the overall performance	Excellent	[43]
Cold-pressed essential oil (CPEO) from lemon and tangerine	No	No (Hexane and dichloromethane)	Yes (Distillation)	No	Yes	Higher carotenoid recovery through azeotropic condensation, adding isopropanol 3.5:1 (ratio) to the CPEO, evaporating under negative pressure, and heating for 2.5 h at 28–32 °C.	Bad	[44]
Lemon (*Citrus limon* cv. *Eureka*) peels, freeze-dried and milled (Ø: ~0.71 mm)	No	Yes (Ethanol–water)	Yes (US)	Yes	Yes	1:40 (*w*:*v*) 75% ethanol, US 550 W for 5 min	Excellent	[45]
Lemon peels, dried and milled (Ø: <0.4 mm)	Yes (Box–Behnken)	No (Hot acidic (HCl) water)	Yes (Temp.)	No	Yes	pH 1.5 at 90 °C for 120 min	Good	[46]
Citrus peel pomace, dried and milled (Ø: 0.177 mm)	No	No (Octenyl succinic anhydride)	Yes (Esterification)	No	Yes	Octenyl succinic anhydride:citrus fiber (1:5, *w*:*w*), pH 8.5 at 20 °C for 1.5 h	Bad	[47]
Lemon peels, dried and milled (Ø: 0.5–3.55 mm)	No	Yes (Deionized water)	Yes (Pressurized)	Yes	Yes	10.34 MPa, rinse volume (30%), purge for 90 s with N_2_ gas, 160 °C for 5 or 30 min (depending on compound)	Excellent	[48]
Lemon peels, freeze-dried and milled (Ø: 0.45 mm)	No	Yes (Sodium acetate buffer and ethanol–water)	Yes (Enzymatic and US)	Yes	Yes	Enzyme (5 U cellulase and pectinase in sodium acetate buffer (20 mM, pH 5.0) at 40 °C for 60 min) and US (400 W, 24 kHz, power level of 50%, 23 °C, 15 min in ethanol:water 50:50) treatments	Excellent	[49]
Lemon processing residues, dried and milled (Ø: <1 mm)	No	Yes (Water, ethanol, and ethanol–water)	Yes (Stirring)	No	Yes	Water as solvent at ratio 1:50, stirring for 30 min	Good	[50]
Lemon peel, dried and milled (Ø: <0.734 mm)	No	Yes (Water and ethanol)	Yes (Stirring)	Yes	Yes	50 g + 500 mL 98% ethanol stirring for 24 h at 25 °C, filtered and concentrated using the vacuum evaporator at 40 °C	Excellent	[51]

Q1: Does the article include an experimental design (response surface methodology)? Q2: Does it use green solvents for the extraction? Q3: Does it use green technologies for the extraction? Q4: Does it extract flavonoids? Q5: Do the authors validate their extract? Response surface methodology, solvent, and extractive technology used are specified in parentheses. Ø: diameter; Temp.: temperature; EOs: essential oils; CO_2_: carbon dioxide; MW: microwave; US: ultrasound; HMIM: hybrid molecularly imprinted membrane; HPH: high-pressure homogenization; PLE-SPE: pressurized liquid extraction coupled in-line with solid-phase extraction.
